# Deep Learning for Dental Diagnosis: A Novel Approach to Furcation Involvement Detection on Periapical Radiographs

**DOI:** 10.3390/bioengineering10070802

**Published:** 2023-07-04

**Authors:** Yi-Cheng Mao, Yen-Cheng Huang, Tsung-Yi Chen, Kuo-Chen Li, Yuan-Jin Lin, Yu-Lin Liu, Hong-Rong Yan, Yu-Jie Yang, Chiung-An Chen, Shih-Lun Chen, Chun-Wei Li, Mei-Ling Chan, Yueh Chuo, Patricia Angela R. Abu

**Affiliations:** 1 Department of General Dentistry, Chang Gung Memorial Hospital, Taoyuan City 33305, Taiwanlynn202207017@jyu.edu.cn (M.-L.C.);; 2 Department of Electronic Engineering, Chung Yuan Christian University, Taoyuan City 32023, Taiwanchischen@cycu.edu.tw (S.-L.C.); 3 Department of Information Management, Chung Yuan Christian University, Taoyuan City 320317, Taiwan; 4 Department of Electrical Engineering and Computer Science, Chung Yuan Christian University, Chung Li City 32023, Taiwan; 5 Department of Electrical Engineering, Ming Chi University of Technology, New Taipei City 243303, Taiwan; 6 School of Physical Educational College, Jiaying University, Meizhou 514000, China; 7 Department of Information Systems and Computer Science, Ateneo de Manila University, Quezon City 1108, Philippines; pabu@ateneo.edu

**Keywords:** deep learning, periapical radiograph, furcation involvement, image segmentation, Gaussian high-pass filtering, image preprocessing, CNN

## Abstract

Furcation defects pose a significant challenge in the diagnosis and treatment planning of periodontal diseases. The accurate detection of furcation involvements (FI) on periapical radiographs (PAs) is crucial for the success of periodontal therapy. This research proposes a deep learning-based approach to furcation defect detection using convolutional neural networks (CNN) with an accuracy rate of 95%. This research has undergone a rigorous review by the Institutional Review Board (IRB) and has received accreditation under number 202002030B0C505. A dataset of 300 periapical radiographs of teeth with and without FI were collected and preprocessed to enhance the quality of the images. The efficient and innovative image masking technique used in this research better enhances the contrast between FI symptoms and other areas. Moreover, this technology highlights the region of interest (ROI) for the subsequent CNN models training with a combination of transfer learning and fine-tuning techniques. The proposed segmentation algorithm demonstrates exceptional performance with an overall accuracy up to 94.97%, surpassing other conventional methods. Moreover, in comparison with existing CNN technology for identifying dental problems, this research proposes an improved adaptive threshold preprocessing technique that produces clearer distinctions between teeth and interdental molars. The proposed model achieves impressive results in detecting FI with identification rates ranging from 92.96% to a remarkable 94.97%. These findings suggest that our deep learning approach holds significant potential for improving the accuracy and efficiency of dental diagnosis. Such AI-assisted dental diagnosis has the potential to improve periodontal diagnosis, treatment planning, and patient outcomes. This research demonstrates the feasibility and effectiveness of using deep learning algorithms for furcation defect detection on periapical radiographs and highlights the potential for AI-assisted dental diagnosis. With the improvement of dental abnormality detection, earlier intervention could be enabled and could ultimately lead to improved patient outcomes.

## 1. Introduction

With the increasing emphasis on health awareness, people are paying more and more attention to health matters. Seeing a doctor or undergoing health check-ups has become part of daily life. However, this has also led to a shortage of medical resources due to the high demand. This research focuses on one of the most high-demand reasons for check-ups, periodontitis. Periodontitis is a type of periodontal disease [1]. The symptoms of periodontitis can be further classified based on the furcation involvements occurring at the bifurcation or trifurcation of the roots of molars. Traditionally, dentists rely on repeated X-ray examinations, palpation, and mobility tests to confirm the presence of furcation involvements (FI) and take appropriate measures [2,3]. Therefore, the purpose of this research is to train a convolutional neural network (CNN) model to accurately identify FI on PAs. This helps dentists to quickly distinguish and compare the severity of the disease, thereby reducing the consumption of medical resources.

The motivation behind this project is to delegate the task of identifying dental symptoms to artificial intelligence (AI). With the rapid development of AI technology, there have been numerous AI applications in recent years, such as vehicle counting [4], financial field applications [5], medical education [6], chip design field [7], and foreign language teaching [8]. In the current standard process of dental diagnosis and treatment, the use of X-rays can reduce the probability of misjudgment by assisting in the identification of symptoms that are difficult to detect with the naked eye. However, there is still a possibility of misjudgment due to differences in lighting or shooting angles. Additionally, dentists spend a significant amount of time interpreting dental lesions before treating each patient, which accumulates into significant time and physical costs for the practitioner. Therefore, a well-trained model from this project could significantly assist dentists in diagnosis. Dentists can use AI-classified images for pre-screening and comparison and then perform further detailed invasive examinations [9]. The goal of this research is to construct a CNN model [10] that can identify the presence or absence of FI [11] through transfer learning. This disease frequently occurs at the root bifurcation of multi-rooted teeth, particularly in upper and lower molars. The key difference between multi-rooted teeth and single-rooted teeth is the number of roots, with multi-rooted teeth resembling a forked root system where the gap between roots is referred to as furcation. Under normal circumstances, the furcation is filled with alveolar bone. However, when periodontal disease occurs, the alveolar bone is lost. Bacteria can penetrate deeper into the gap, ultimately leading to a decrease in tooth stability or even tooth loss. The prevalence of periodontal disease today is a common occurrence that is also associated with an increased incidence of FI [12]. Since FI usually occurs in narrow and complex-to-clean locations, missing the golden treatment period can quickly escalate the disease to a point where even surgery cannot restore long-term stability [13]. The early detection and repair of bone augmentation can prevent tooth loss.

Radiographic diagnosis is the most widely used and important means of evaluating teeth in dentistry. The use of new X-ray techniques like cone beam-computed tomography (CBCT) and magnetic resonance imaging (MRI) has the potential to enhance the accuracy of diagnosing root canal bifurcations [14]. This means that clinical dentists still mostly rely on traditional X-ray images. Although these new imaging techniques are indeed more precise than traditional 2D X-rays in many areas, they are still not widely used in general clinical practice due to the time and cost required. Additionally, the resources for these techniques are limited and difficult to distribute equitably to patients. Furthermore, high-precision images like those provided by CBCT are only helpful in assisting dentists with initial diagnoses [15,16]. Unless the condition is complex, dentists still rely more on traditional X-rays such as PA, bitewing, and panoramic films. The main goal of this research is to address the shortcomings of traditional X-ray images and improve image quality by reducing noise or improving clarity. Additionally, this research aims to assist or simplify the clinical workflow for dentists by training a CNN transfer learning model to automatically detect and identify periapical lesions on PA images. This will save dentists time and energy in reviewing PA images and reduce the risk of visual fatigue [2,17]. Moreover, the model will better define FI lesions and eliminate the need for the discussion or repeated confirmation of suspicious lesions [18,19,20,21]. This helps dentists to reduce patient consultation time and respond more quickly to these elusive conditions. The Innovations of this research are listed as follows:

1. A CNN-based automated recognition system for FI lesions has been developed in this research, and the proposed final model can achieve an accuracy of 94%, which is a 5% increase compared to [19].
2. An adaptive threshold and an adjusted segmentation line operation have been proposed in this research to enhance fault tolerance, which has proven helpful for the research process and final learning results.
3. The model for distinguishing between single-rooted and double-rooted teeth in this research has achieved a high recognition accuracy of 97%, which enables the proper classification of the sample data contained in a single image. Additionally, the proposed model for classifying single and double-rooted teeth can help in the collection and categorization of samples for medical and AI automation applications in the future.

The structure of this research is as follows: Section 2 introduces the CNN model architecture and the automated image data generation methods used for training. Section 3 presents and analyzes the results of various experiments, including comparisons between different models and an examination of factors that may have impacted the outcomes. Section 4 discusses the findings obtained from the experiments. Finally, Section 5 concludes this research and suggests future directions for further explorations.

## 2. Materials and Methods

**Figure 1:**
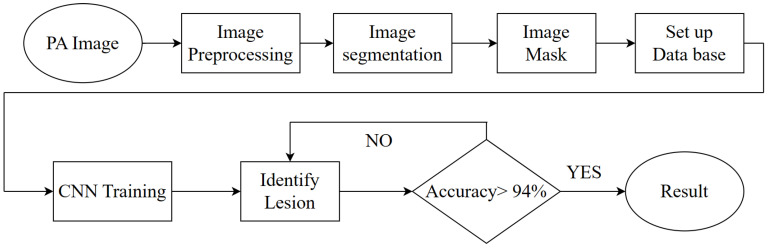
The flowchart of this proposal.

In this research, the most important areas are image preprocessing and image masking, which were the main factors affecting CNN training and validation. In the image preprocessing step, the noise in the original PA image is removed. In the meantime, the characteristics of the diseases classified in this research can be enhanced. This step is crucial to the next PA image classification step as it obtains better recognition accuracy. The overall flow chart of this research is shown in Figure 1. 

### 2.1. Image Preprocessing

**Figure 2:**
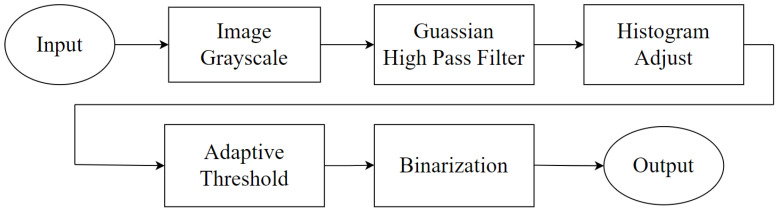
The flowchart of using this system.

One of the focuses of this research is to locate FI in the posterior molars from PA images. However, due to issues such as the angle of the X-ray beam or lighting, distinguishing the three targets in PA images (teeth, gingiva, and background) is often challenging. Additionally, PA images frequently contain noise and distortion, which make it tedious and time-consuming for dentists to locate the targets and might cause the possibility of misdiagnosis. Therefore, the aim of this step is to standardize PA images by eliminating interfering confounding variables and enabling the clear differentiation of the three targets. The pre-processing step comprises three parts: gray-level adjustment, Gaussian high-pass filtering, and adaptive thresholding, as shown in Figure 2. 

#### 2.1.1. Image Grayscale

To improve the image adjustment and the efficiency of CNN training, the original RGB images are converted to grayscale images. While RGB images have three dimensions, grayscale images have only two dimensions and are more suitable for image adjustment. Furthermore, since the colors captured by PA images are grayscale, there is no loss of information in converting to grayscale [22]. This conversion simplifies the representation of the image data and allows the pixel coordinates of the image to be more easily displayed. Grayscaling makes it simpler to detect errors and make adjustments.

#### 2.1.2. Gaussian High-Pass Filtering

**Figure 3:**
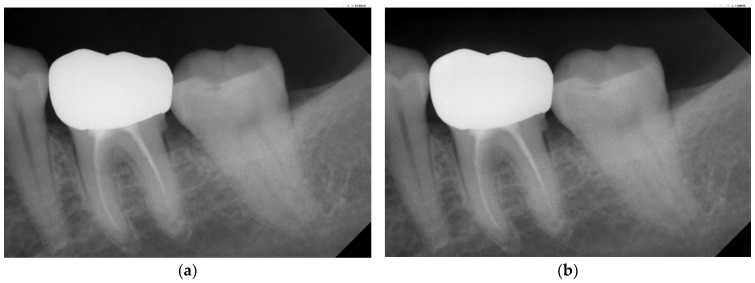
Schematic diagram of the result of Gaussian filtering on the image. (**a**) Original image. (**b**) Using Gaussian high-pass filter.

The most challenging problems in this research are the image noise on the PA image and the indistinct contours of the target disease. Cui and Zhang [23] used frequency domain filtering to sharpen the image in which the edge features are highlighted. Gaussian filtering is separated into high-pass and low-pass filtering. Low-pass filtering can filter out the noise. It is concentrated in high frequencies and smooths the image edge, but it can also cause the image to become too blurry and lose details. On the other hand, high-pass filtering can suppress the low-frequency parts and focus on highlighting the edge features, effectively extracting noise and interference. Thus, this research subtracts the filtered noise image from the original image, as described in [24]. Equation (1) can decrease the noise and interference on the original image. Figure 3 shows the results of achieving a more pronounced contrast, displaying different gray levels in different areas and clearer tooth contours.

(1) $$H\left(u,\;v\right)=1-e^{\frac{-D^{2}\left(u,\;v\right)}{2D_{0}^{2}}}$$

#### 2.1.3. Adaptive Threshold

After filtering out the noise and highlighting the contours of PA images, the adaptive thresholding is performed. The main goal of this step was to find a suitable threshold for the image to perform binarization, dividing the image into two parts: teeth and gums, background and diseases, and alveolar bone. The accuracy of this step affected the determination of the target object in the later steps. This research tested the fixed threshold using the Otsu algorithm, as mentioned in [25]; the iterative algorithm, as mentioned in [26]; and the adaptive threshold, as mentioned in [27]. However, for the molars’ PA, the pixel brightness was a significant factor, which is different from the PA of a single tooth. The variation in the molar area makes it even more challenging to find a pattern. Therefore, this research developed a newly defined adaptive algorithm to find the optimal threshold.

**Figure 4:**
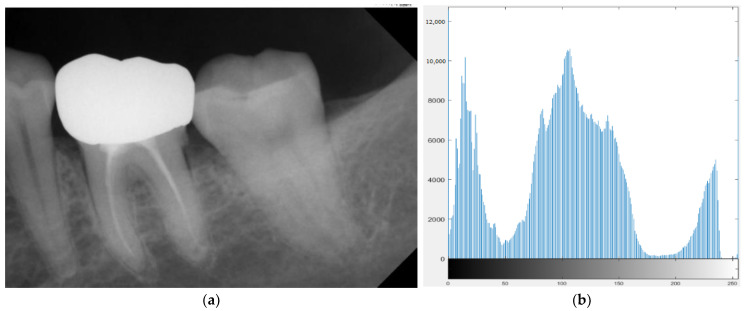

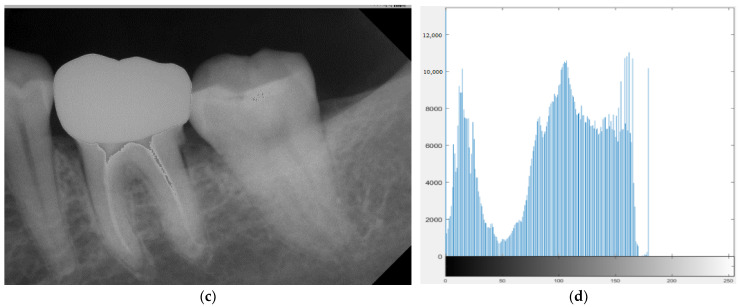
The result of the adaptive threshold. (**a**,**b**) are the original image and histogram. (**c**,**d**) adjust the grayscale and histogram.

To address the issue of possible extreme values in the images, Chen et al. adjusted the grayscale image to avoid this problem [28]. Based on that, this research improved the process by redistributing extremely bright areas (grayscale > 170) to lower grayscale. After the adjustment, the subsequent algorithms were not affected by external lighting factors during the image capture process. The result is shown in Figure 4.

After solving the extreme value variations, adaptive threshold values could be calculated. First, the minimum value (*Zmin*) in the grayscale range of 60 to 120, the maximum value (*Lmax*) in the grayscale range of 30 to 90, and the maximum value (*Rmax*) in the grayscale range of 91 to 170 was identified from the preprocessed grayscale image. Second, the midpoint gray value (*Zmid*) was calculated using Equation (2) and used as the initial binary threshold value (T0). In the next step, the total number of pixels in the image (*Ztotal*) and the total number of pixels from T0 to 170 (*Zcheck*) was calculated to obtain all parameters. Finally, three verification methods, namely checking whether the X-distance between two pairs of values (*Zmin*, *Zmid*) was less than 15, Equation (3), and Equation (4), were utilized to validate the results. If any of these tests failed, the process entered into an iterative calculation, either by changing the first step to find the second lowest value or by adjusting the threshold value to meet the restrictions.

(2) $$Z_{mid}=\frac{Lmax+Rmax}{2}$$

(3) $$Z_{check}\leq \;Z_{total}\times \frac{5}{6}$$

(4) $$Z_{check}\geq \;Z_{total}\times \frac{2}{3}$$

**Figure 5:**
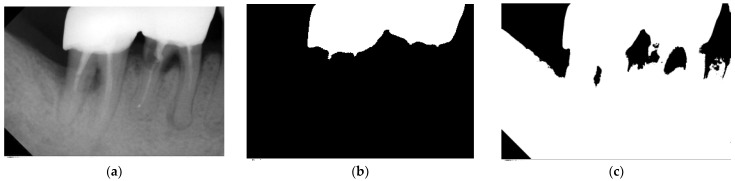
The result of the preprocessing. (**a**) Original image. (**b**) Directly binarized image. (**c**) Adjusted grayscale image.

Two situations require the re-finding of the threshold. The first is when multiple T0 values meet the above constraints, and the other is when the suitable threshold within the grayscale range of 80–95 cannot be found. These two situations may cause multiple unsatisfactory segmenting results in the image cropping stage. Therefore, the ideal threshold value is continuously re-found through an iterative method. The ideal threshold value is used for binary thresholding where the pixel value greater than the threshold value is set to 1 (white) and the other pixel values are set to 0 (black). The binary image result was tested to ensure that the total mean of all pixels is greater than 0.6 and less than 0.85. The binary result is shown in Figure 5.

### 2.2. Image Segmentation

The purpose of this step is to separate each tooth in PA image and create a database of images for each tooth. This step can effectively improve the target object recognition and reduce the interference from non-target objects before CNN training.

This research tested the segmentation method proposed in previous research [27]. The result showed that it worked well for front teeth but had difficulty with back teeth due to lighting or imaging conditions. Therefore, this research modified the method based on the other research [29] to automatically locate the segmenting line for back teeth. The masking technique for CNN training was also adjusted. The details of these modifications are described in the following sections.

#### 2.2.1. Vertical Projection

**Figure 6:**
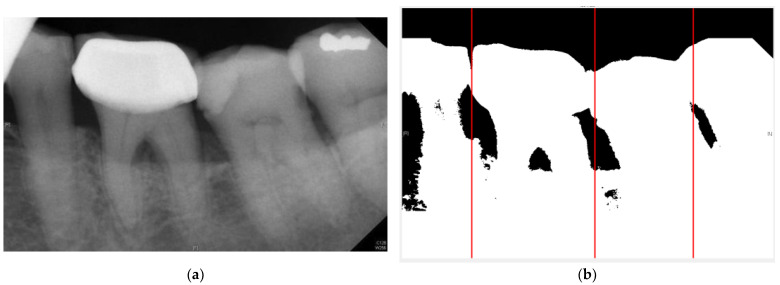
The segmenting line results for this study. (**a**) Original image (**b**) Preliminary cutting line coordinates. The tangent line is shown as the red line.

Neighboring teeth segmenting lines inevitably lie on the interdental space, which is black (pixel value 0) in the binary image of PA. In addition, a PA image can have up to five teeth, so this research calculated the vertical pixel sum of each row. According to the algorithm conditions, neighboring interdental spaces must be divided by at least one tooth distance. The result is shown in Figure 6.

#### 2.2.2. Rote Tangent

**Figure 7:**
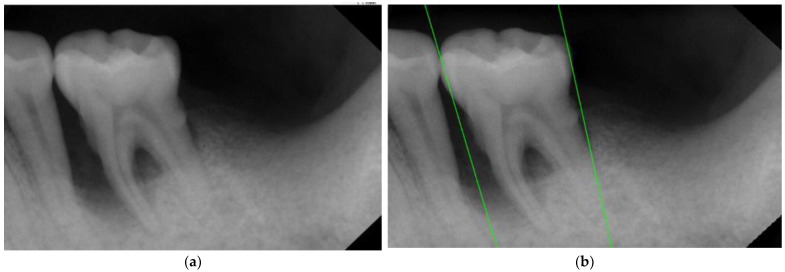
The result image of segmenting lines. (**a**) Original image. (**b**) After rotating segmentation lines. Corrected tangents are shown as green lines.

Unlike the rotation algorithm in other research [29], instead of rotating the image, this research moves the vertical coordinates to rotate the segmenting line and create the marked positions. The positions of the five smallest pixel values are marked from left to right. This operation can avoid encountering complex rotation functions and converting the image coordinate system to or from the original coordinate system. It makes the automated program simpler, more efficient, and more error-tolerant. The segmentation result is shown in Figure 7. After locating the optimal rotation for the segmentation line, the coordinates of the two endpoints of the segmentation line are obtained. Comparing the distances between the two endpoints and the target tooth, the endpoint which is further away from the target tooth is considered to be on the outer side of the tooth. Then, a vertical trimming is performed on the X-coordinate to ensure that the target tooth is included without cutting through the tooth root. Bad segmentation would cause the loss of features. Previous research [27] has proposed using grayscale for segmentation. However, for posterior periapical radiographs (PA), which are sensitive to lighting conditions, the high grayscale values of gingiva can be similar to or even higher than those of the tooth roots. This can lead to misjudgment during the subsequent rotation and segmentation steps, indirectly confirming the importance of the pre-processing step mentioned.

### 2.3. Image Mask

After determining the optimal rotation angle and segmentation lines, automated masking was applied to the areas outside the two segmentation lines. This isolates the target object of interest (a single tooth) from external factors that may affect the accuracy of CNN recognition and can improve the learning effectiveness of CNN in recognizing the target object. 

**Figure 8:**
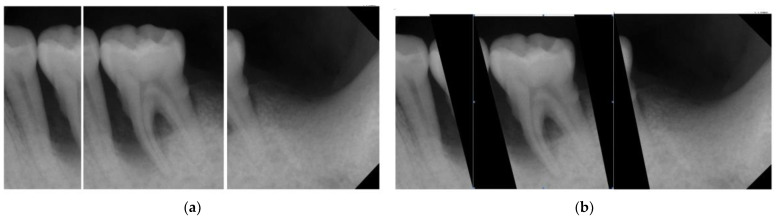
The results of the masking image. (**a**) Original segmentation. (**b**) Retouched segmentation and masking.

This research proposes a method expanding each cutting line outward by 1/30th of the original image width to avoid damaging the target while the misplacement of the segmentation lines occurs. This step provides some error tolerance to the process. In addition, the extended area provides surrounding information of the target object that would help with CNN training. The segmentation result is shown in Figure 8.

### 2.4. Image Identification

To validate the effectiveness and reliability of the proposed model, this study selected 128 lesion images and 140 normal teeth images from the database, as listed in Table 1. The images are augmented through horizontal, vertical, and reverse flipping to increase the number of images. Based on transfer learning theory, the database was divided into training and validation sets in a ratio of approximately 7:3 and was classified into the database, as shown in Table 2.

#### 2.4.1. CNN Model

The experimental environment used in this proposal includes hardware and software specifications as shown in Table 3. Several famous transfer learning models in Matlab namely GoogLeNet, AlexNet, Inception v3, and Vgg19 are used for comparison. Taking GoogLeNet, which was performed best in this experiment as an example, the architecture is shown in Table 4. GoogLeNet is composed of Inception modules [30], which allow GoogLeNet to obtain the kernels of different scales during training and learn multiple features. Additionally, the inclusion of 1 × 1 convolutional layers prevents an excessive number of kernels and increases the non-linearity of the neural network with more comprehensive learning. Moreover, GoogLeNet eliminates connected layers. It reduces the number of parameters by nearly nine times compared to AlexNet [31]. Despite achieving similar or even higher accuracy than other models, the significant reduction in parameters makes GoogLeNet much lighter compared to other models. The remaining two models used for the experiment are Vgg19 [32] and Inception v3 [33]. These two models have shown better performance than other image recognition models in detecting image patterns. 

The randomly selected validation dataset is tested for the proposed model after the transfer learning is accomplished. The validation accuracy is then calculated and evaluated. The confusion matrix can be calculated to evaluate the quality of the trained model.

#### 2.4.2. Adjust Hyper-Parameter

The adjustment of hyper-parameters is crucial for deep learning outcomes. The best combination of parameter settings can be slowly found through the appropriate fine tuning for each training process. The most frequently adjusted parameters in this experiment are max epoch, initial learning rate, mini batch size, and learn drop period. The suggested values of hyper-parameters are shown in Table 5.

A. Optimizer

SGDM (stochastic gradient descent with momentum) and Adam (adaptive moment estimation) are two popular optimization algorithms used in deep learning to train neural networks. Although Adam is faster than SGDM in terms of training speed, SGDM exhibits better convergence and more stable training performance. Considering the current number of images in the training set, the advantage of using Adam’s fast convergence speed is not significant and may encounter convergence issues.

B. Initial Learning Rate

The rate at which the gradient descends during model training is affected by the initial learning rate. A small value can cause slow convergence and make the model prone to overfitting. Conversely, a large value can cause the model to learn too quickly and fail to converge, leading to divergence. After several trials, a stable learning rate of 1e-4 was determined for GoogLeNet.

C. Mini Batch Size

The mini batch size parameter determines how many data points are used to train the neural network at once. It is essentially a subset of the training set. If the mini batch size is too large, more data need to be considered for training. This leads to a more accurate correction direction, but the training process will take longer. On the other hand, if the mini batch size is too small, the correct direction will be biased because only a small amount of data are used in each iteration. However, this allows for more frequent corrections. For example, if the mini batch size is set to 20, this means that only 20 data points are used for training at a time. The mini batch size and epoch are closely related. If there are 400 data points in total and mini batch size is set to 20, then 20 training instances comprise one epoch.

## 3. Results

**Figure 9:**
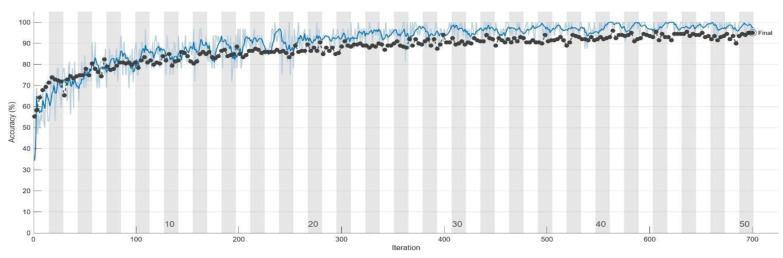
The accuracy of GoogLeNet model during training process. The validation set is the black line and the training set is the blue line.

**Figure 10:**
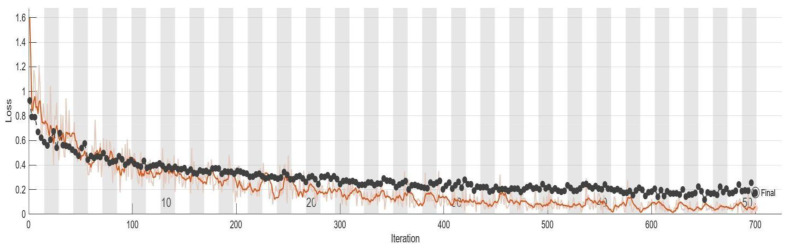
The loss of GoogLeNet model during training process. The validation set is the black line, and the training set is the orange line.

This section provides an overview of the model performance in this research. To monitor the training progress, a validation set was utilized. Table 6 presents the training process of GoogLeNet at intervals of five epochs. Additionally, Figure 9 and Figure 10 offer a detailed representation of the training progress of GoogLeNet, including the final convergence status. The black line in both figures represents the validation results. Finally, the trained model was tested using the test set, and the confusion matrix was calculated. The results of the confusion matrix are presented in Table 7.

**Figure 11:**
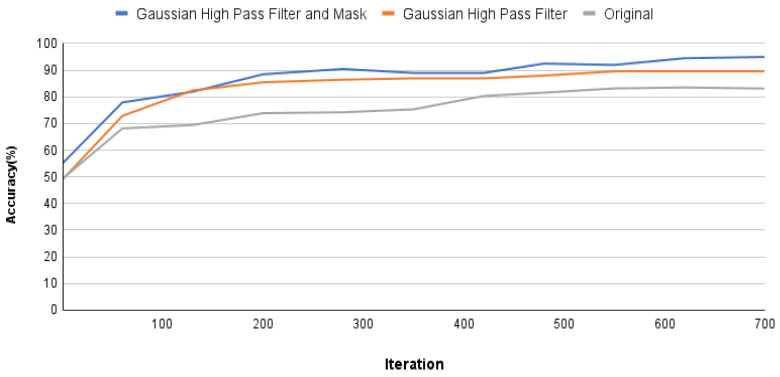
Accuracy comparison of GoogLeNet training process using only Gaussian high-pass filter and additional high-precision masking.

Based on the data presented in Table 8, it is evident that using PA images without excessive noise adjustment as a training database leads to an accuracy of over 80%. However, this approach also results in significant loss on the validation set. These findings suggest potential flaws in the database, such as blurred features or excessive noise. The second column demonstrates the results of training with Gaussian high-pass filtered raw images. Correcting image size and enhancing features significantly reduces the loss, resulting in an accuracy of 87.21%. However, these results fall short of the project’s standards. Additionally, the loss rebounds after reaching 0.4 during training, indicating the need for further image preprocessing. The third column of Table 8 showcases the results of this project, which involve enhancing image features through masking techniques to exclude non-target regions. This enhancement dramatically improves the model’s performance in image classification, achieving a validation set accuracy of 94.97% and reducing the loss to below the threshold of 0.18. Furthermore, Figure 11 illustrates the training process using different image preprocessing techniques. The three curves represent test accuracy on the training set. All curves show an increasing trend in accuracy as the number of iterations increases. The gray curve represents post-training using raw images. The orange curve represents applying high-precision automatic segmentation to raw images, followed by a Gaussian high-pass filter. The blue curve incorporates the previous process with an automatic masking step. The trend of the line graph indicates the significant impact of image preprocessing on the accuracy, further demonstrating that adding image filters and masking processing can significantly improve the model’s accuracy, with an improvement rate as high as 10.8%.

**Figure 12:**
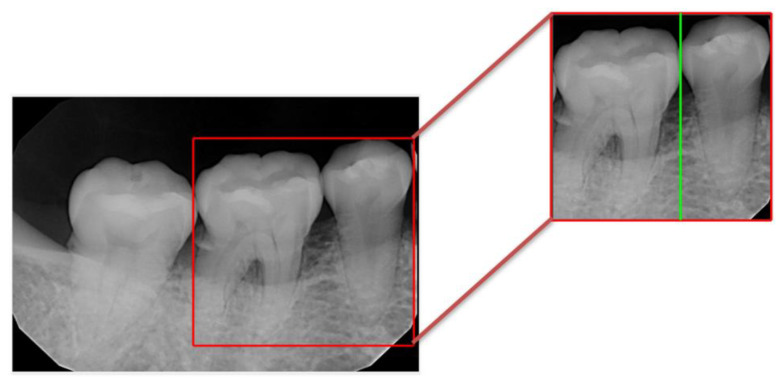
Validation example after image cropping in this study.

Classifying images into molars and non-molar teeth was the first trial in this research, as illustrated in Figure 12, where the molar tooth on the left was the target, and the single-rooted non-molar tooth on the right was used as a comparison. A CNN model was developed for this classification task. The results demonstrated excellent classification accuracy with an average of over 97.5%, as shown in Table 9. 

To enhance the recognition accuracy of the model on PA images for FI, the training samples were filtered to focus on the variables that could affect recognition accuracy. This approach made CNN more sensitive to the disease, more focused on the target, and resulted in a higher learning effect. In this study, image screening was performed on the training samples to improve the recognition accuracy. The results in Table 10 indicate that the CNN classification had an impact of 3–4% on the training outcomes. Moreover, in order to assess the model’s performance, a set of evaluation metrics was employed, including recall, precision, and F1 score. These metrics provide a comprehensive analysis of the model’s ability to accurately classify various cases and identify relevant features. Furthermore, alongside evaluating the model’s performance, it is crucial to analyze the computational aspects of the proposed method. This analysis incorporates metrics such as computation time and actual operating time. These metrics facilitate a comparison of the efficiency and scalability of the proposed methods and aid in understanding the practical feasibility and potential computational requirements of the approach. The findings are presented in Table 11.

Table 12 indicates that the automated FI detection results in this research exhibit a significant contrast to the disease identification accuracy obtained using CT images at the apical region in the literature [34]. The symptom judgment accuracy using Vgg19 was nearly 93%, while the judgment accuracy of GoogLeNet and AlexNet was nearly 95% in this research. 

## 4. Discussion

A preprocessing step for dental images was found to be crucial in the research process. Proper preprocessing is essential for training a CNN, as raw PA images may not provide accurate information without it. However, establishing a standard processing method applicable to all images is challenging due to interference and external factors unique to each image. A lack of preprocessing can make subsequent segmentation difficult and result in lower accuracy due to potential noise in the PA images. In this study, the accuracy of the trained model was significantly improved by segmenting multiple teeth in PA images into individual tooth images before training. The adaptive threshold preprocessing method designed in this study accurately defined the cutting points during image segmentation, leading to improved segmentation accuracy. Preprocessing techniques like Gaussian high-pass filtering also reduced the inclusion of non-target regions. These improvements in segmentation accuracy enhanced symptom enhancement and overall model accuracy, highlighting the importance of preprocessing in this study. Furthermore, an automated process was developed to assist dentists in identifying bifurcations in PA images without causing visual or mental fatigue. During the CNN training phase, several mainstream image recognition models were tested, all achieving judgment accuracies above 90%. Following preprocessing and initial CNN recognition, the model effectively located teeth with potential diseases, with accuracy comparable to visual judgment. Specifically, GoogLeNet and AlexNet achieved judgment accuracies close to 95%. In comparison to the method proposed in [34], this study improved judgment accuracy by nearly 5%. These results demonstrate the efficacy of the proposed technique in detecting FI disease and showcase the success of training a CNN using conventional PA images, surpassing recognition capabilities based on CT images.

The results indicate that the proposed model exhibits mature and successful problem-solving capabilities, producing results highly similar to human judgment. However, there is room for improvement in the model’s ability to further classify FI disease locations and enhance existing image enhancement techniques. In the future, this research aims to improve the automated process flow by integrating it within a chip, augmenting databases and developing a GUI interface. This will allow the automated process flow to be successfully integrated into clinical operations for dentists and result in a reduction in their workload and a shortening of patients’ treatment time.

## 5. Conclusions

In general, this research study highlights the importance of preprocessing in improving the accuracy of a CNN model for detecting furcation involvements (FI) in dental images. The findings demonstrate that without proper preprocessing, raw PA images do not provide accurate information for training the model. The study proposes various preprocessing techniques, such as adaptive thresholding and Gaussian high-pass filtering, which significantly enhances the segmentation accuracy and the overall performance of the model. Additionally, an automated process was developed to assist dentists in identifying FI in PA images, offering a reliable and efficient alternative to visual judgment. The trained CNN model, particularly utilizing GoogLeNet and AlexNet architectures, achieved high accuracy in locating teeth with potential diseases, surpassing the performance of previous methods. Overall, this study provides valuable insights into the significance of preprocessing and the potential of CNN models in dental image analysis. The results contribute to the development of a high-accuracy medical assistance system, reducing the workload for dentists and improving the quality of dental care. Further research can build upon these findings to refine the model and explore additional enhancements for accurate FI detection and classification.

## Figures and Tables

**Table 1:** Data classification of original periapical image from clinical.

The Number of Original Images from Clinical			
Tooth	Lesion	Normal	total
Quantity	128	140	368

**Table 2:** Data classification of periapical image after preprocessing.

	Training Set	Validation Set	Total
Lesion	271 (Expanded)	41	312
Normal	245 (Expanded)	106	351

**Table 3:** The hardware and software detailed specifications.

Hardware Platform	Version
CPU	AMD R5-5600X
GPU	GeForce GTX 1660 SUPER
DRAM	DDR4 3200 32 GB
OS	Windows 10
Software platform	Version
MATLAB	R2022b

**Table 4:** The input and output of GoogLeNet model.

Layer	Type	Activation
1	Data	224 × 224 × 3
2	Convolution	112 × 112 × 64
3	Max Pool	56 × 56 × 64
4	Convolution	56 × 56 × 192
5	Max Pool	28 × 28 × 192
6	Inception (3a)	28 × 28 × 256
7	Inception (3b)	28 × 28 × 480
8	Max Pool	14 × 14 × 480
9	Inception (4a)	14 × 14× 512
10	Inception (4b)	14 × 14 × 512
11	Inception (4c)	14 × 14 × 512
12	Inception (4d)	14 × 14 × 528
13	Inception (4e)	14 × 14 × 832
14	Max Pool	7 × 7 × 832
15	Inception (5a)	7 × 7 × 832
16	Inception (5b)	7 × 7 × 1024
17	Avg Pool	1 × 1 × 1024
18	Dropout (40%)	1 × 1 × 1024
19	Linear	1 × 1 × 1000
20	Softmax	1 × 1 × 1000

**Table 5:** This study uses hyperparameters in the CNN model.

Hyperparameters	Value
Max Epoch	50
Initial Learning Rate	0.0001
Mini Batch Size	32
Learn Drop Period	5
Validation Frequency	3
Learn Rate Drop Factor	0.2000

**Table 6:** The training process of GoogLeNet with every five epochs as the unit period.

Epoch	Iteration	Time Elapsed	Mini-Batch Accuracy	Validation Accuracy	Mini-Batch Loss	Validation Loss
1	1	00:00:02	34.38%	55.28%	1.6001	0.9257
5	60	00:00:37	90.62%	77.89%	0.3410	0.4747
10	130	00:01:15	75.00%	81.91%	0.5390	0.3947
15	200	00:01:53	78.12%	88.44%	0.3817	0.3297
20	280	00:02:40	87.50%	90.45%	0.2633	0.2445
25	350	00:03:17	93.75%	88.94%	0.1366	0.2721
30	420	00:03:55	96.88%	89.95%	0.0668	0.2637
35	480	00:04:28	90.62%	92.46%	0.1284	0.2018
40	550	00:05:07	93.75%	91.96%	0.1689	0.2093
45	620	00:05:44	93.75%	94.47%	0.1187	0.1702
50	700	00:06:30	96.88%	94.97%	0.0486	0.1700

**Table 7:** The confusion matrix of the GoogLeNet training result.

		Actual Values	
		Normal	Lesion
Predicted Value	Normal	46.8%	4.3%
	Lesion	2.1%	46.8%

**Table 8:** Compare the impact of various training sets on training results.

	Original Images	Gaussian High-Pass Filter	Gaussian High-Pass Filter + Mask
Validation Accuracy	84.16%	87.21%	94.97%
Validation Loss	0.7634	0.4578	0.1822
Model	GoogLeNet	GoogLeNet	GoogLeNet
Image	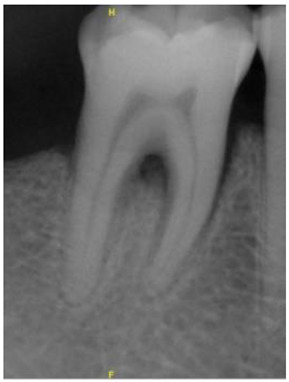	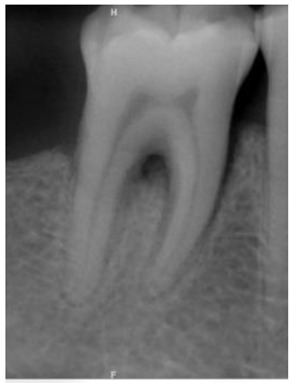	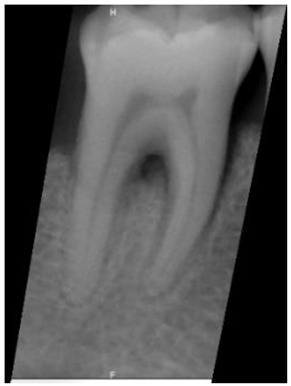

**Table 9:** The clinical data compare to the result.

Tooth Position in Figure 12	Recognition Accuracy	
Clinical Analysis	Molar	Single Tooth
Vgg19	98.01%	97.53%
Inception v3	97.76%	98.01%
Google Net	98.51%	98.42%
AlexNet	98.51%	98.26%

**Table 10:** Result of the FI identification accuracy of different models.

Training Process	Directly Identify the Disease	Identify the Disease after Classification
Vgg19	89.21%	92.96%
Inception v3	91.58%	94.23%
Google Net	92.18%	95.48%
AlexNet	91.58%	94.97%

**Table 11:** Model Efficacy Comparison For FI.

	GoogLeNet	Vgg19	AlexNet	Inceptionv3
Accuracy	94.97%	92.96%	94.92%	94.21%
Recall	95.6%	73.9%	86.9%	86.9%
Precision	91.6%	68%	80.0%	83.3%
F1	93.5%	70.8%	83.3%	85.0%
Elapsed time	25 min 30 s	87 min 47 s	29 min 34 s	76 min 50 s
Runtime	2.5981 s	6.4115 s	2.7535 s	4.2417 s

**Table 12:** Comparison results of CNN models in this study and past studies.

	Method in [34]	This Research			
		GoogLeNet	AlexNet	Vgg19	Inceptionv3
Accuracy	89%	94.97%	94.92%	92.96%	94.21%
